# UVSSA, UBP12, and RDO2/TFIIS Contribute to Arabidopsis UV Tolerance

**DOI:** 10.3389/fpls.2019.00516

**Published:** 2019-04-24

**Authors:** Wesam M. Al Khateeb, Annan A. Sher, Jeffery M. Marcus, Dana F. Schroeder

**Affiliations:** ^1^Department of Biological Sciences, Yarmouk University, Irbid, Jordan; ^2^Department of Biological Sciences, University of Manitoba, Winnipeg, MB, Canada

**Keywords:** Arabidopsis, transcription coupled repair, UV, CSA, CSB, UVSSA, UBP7, TFIIS

## Abstract

Plant DNA is damaged by exposure to solar radiation, which includes ultraviolet (UV) rays. UV damaged DNA is repaired either by photolyases, using visible light energy, or by nucleotide excision repair (NER), also known as dark repair. NER consists of two subpathways: global genomic repair (GGR), which repairs untranscribed DNA throughout the genome, and transcription-coupled repair (TCR), which repairs transcribed DNA. In mammals, CSA, CSB, UVSSA, USP7, and TFIIS have been implicated in TCR. Arabidopsis homologs of CSA (AtCSA-1/2) and CSB (CHR8) have previously been shown to contribute to UV tolerance. Here we examine the role of Arabidopsis homologs of UVSSA, USP7 (UBP12/13), and TFIIS (RDO2) in UV tolerance. We find that loss of function alleles of *UVSSA, UBP12*, and *RDO2* exhibit increased UV sensitivity in both seedlings and adults. UV sensitivity in *atcsa-1, uvssa*, and *ubp12* mutants is specific to dark conditions, consistent with a role in NER. Interestingly, *chr8* mutants exhibit UV sensitivity in both light and dark conditions, suggesting that the Arabidopsis CSB homolog may play a role in both NER and light repair. Overall our results indicate a conserved role for UVSSA, USP7 (UBP12), and TFIIS (RDO2) in TCR.

## Introduction

Unable to move, plants must adapt to their surroundings. An important and unavoidable component of a plant’s environment is solar radiation, which includes both beneficial visible light and damaging ultraviolet (UV) rays. UV radiation harms a variety of cellular components including DNA. UV damaged DNA, primarily pyrimidine photodimers, is repaired by photolyases, using the energy from visible light (light repair), and by nucleotide excision repair (NER) (dark repair) (Pang and Hays, 1991; Molinier, 2017).

Nucleotide excision repair is a conserved multistep pathway involving damage recognition, strand unwinding, excision, repair synthesis, and ligation. Damage recognition is via one of two NER sub-pathways. Global genomic repair (GGR) identifies UV damage in DNA throughout the genome, while transcription coupled repair (TCR) initiates repair of transcribed strands. TCR has been well studied in humans, where deficiencies in this process can result in Cockayne Syndrome and UV-sensitive syndrome (Gregersen and Svejstrup, 2018). UV damaged DNA arrests progression of RNA polymerase II (RNAP), resulting in stabilization of RNAP – Cockayne Syndrome B (CSB) interaction. CSB then recruits the Cockayne Syndrome A (CSA)-DDB1-cullin 4 complex, which ubiquitinates CSB, followed by UV Stimulated Scaffold protein A (UVSSA) and Ubiquitin Specific Peptidase 7 (USP7), which stabilize CSB. Subsequently, core NER components, such as TFIIH and the XPG and XPF endonucleases, are recruited, and resulting in damage excision and repair. Re-initiation of transcription following repair is thought to involve the TFIIS elongation factor (Geijer and Marteijn, 2018).

In plants, UV damage in transcribed strands is preferentially repaired, and this process is regulated by the circadian clock (Fidantsef and Britt, 2012; Oztas et al., 2018). The Arabidopsis homologs of CSA, CSB, USP7, and TFIIS have previously been identified and described. *Arabidopsis thaliana* has two CSA homologs, AtCSA-1/ CSAat1A (At1g27840) and AtCSA-2/ CSAat1B (At1g19750) (Kunz et al., 2005). Despite the fact that these two proteins are 92% identical, they are both required for tolerance to UV and MMS and repair of transcribed strands. The CSA homologs interact with DDB1A, localize to the nucleus, and form heterotetramers (Biedermann and Hellmann, 2010; Zhang et al., 2010). The Arabidopsis CSB homolog is SWI2/SNF2 protein Chromatin Remodeling 8 (CHR8, At2g18760) (Kunz et al., 2005; Singh et al., 2010). *CHR8* RNAi lines result in UV sensitivity, but do not exhibit ionizing radiation or intrachromosomal recombination rate phenotypes, consistent with a role in NER (Shaked et al., 2006). UBP12 (At5g06600) and UBP13 (At3g11910) are the Arabidopsis USP7 homologs and have been implicated in plant immunity, flowering, seed, and root development, as well as jasmonate signaling (Ewan et al., 2011; Cui et al., 2013; Derkacheva et al., 2016; Jeong et al., 2017; An et al., 2018). The Arabidopsis TFIIS homolog is Reduced Dormancy 2 (RDO2, At2g38560), which is required for regulation of seed dormancy by *Delay of Germination 1 (DOG1)* (Léon-Kloosterziel et al., 1996; Grasser et al., 2009; Liu et al., 2011; Mortensen and Grasser, 2014). RDO2/TFIIS has also been implicated in mRNA processing in plants, including in response to light (Dolata et al., 2015; Antosz et al., 2017; Godoy Herz et al., 2019). In this study we identify the Arabidopsis UVSSA homolog and examine the roles of UVSSA, UBP12/13, and RDO2 in UV tolerance.

## Materials and Methods

### Phylogenic Tree Construction

Gymnosperm UVSSA homologs were accessed via the PLAZA gymnosperm site^1^ (Proost et al., 2015) while all other homologs were identified via KEGG (Kyoto Encyclopedia of Genes and Genomes^2^). UVSSA amino acid sequences were aligned in CLUSTAL Omega (Sievers et al., 2011) using the default settings and saved in NEXUS format for phylogenetic analysis. The aligned amino acid sequences were then analyzed by maximum parsimony as implemented in PAUP^∗^ version 4.0b8/4.0d78 using the default settings unless otherwise specified (Swofford, 2002). One million maximum parsimony heuristic search replicates were performed with random sequence addition, tree bisection and reconnection branch swapping on only the best trees, multiple trees saved at each step, and retention of all best trees. In addition, 1 million random sequence addition fast addition bootstrap search replicates were performed with retention of all groups consistent with 50% bootstrap consensus.

### Plant Material and Growth Conditions

The following T-DNA alleles were used in this study: SALK_030558 (*AtCSA-1*) (Lee et al., 2010), SALK_000799 and SAIL_273_G11 (*CHR8*), SAIL_58_C12 and SALK_061538 (*UVSSA*), GABI_742C10 (*UBP12*) (Cui et al., 2013), and SALK_027259 (*RDO2*) (Grasser et al., 2009; Liu et al., 2011). Col-0 was used as the wild type control for the SALK and GABI lines (Alonso et al., 2003; Kleinboelting et al., 2012), while Col-3 was used as the control for the SAIL lines (Sessions et al., 2002). All plant material was obtained from the Arabidopsis Biological Resource Center (ABRC) (Columbus, OH, United States) or the Nottingham Arabidopsis Stock Centre (NASC) (Nottingham, Loughborough, United Kingdom). Alleles were genotyped with the primers listed in Supplementary Table S1 along with T-DNA specific primers LBb1.3: ATTTTGCCGATTTCGGAAC (SALK lines), LB3SAIL: TAGCATCTGAATTTCATAACCAATCTCGATACAC (SAIL lines), and GK_8409: ATATTGACCATCATACTCATTGC (GABI line). For plant growth, seeds were sterilized and plated on Linsmaier and Skoog (LS) media (Caisson, Smithfield, UT, United States) with 0.6% sucrose and 0.8% Phytoblend (Caisson). After 2–3 days of stratification at 4°C, plates were moved to an incubator with fluorescent bulbs (100 μM photons m^-2^ s^-1^) and grown under long day conditions (16 h light/8 h dark) at 20°C and 50% relative humidity. For adult growth, 14 day old plants were transplanted into soil (Sunshine mix no. 1, Sun Gro, Bellevue, WA, United States) and grown under the same conditions.

### RNA Extraction and RT-PCR

Ribonucleic acid was extracted from approximately fifty 7-day-old seedlings per genotype with the RNeasy plant mini kit (Qiagen, Hilden, Germany) according to the manufacturer’s instructions including a DNase treatment. RNA was quantified with a Nanodrop spectrophotometer (Thermo Scientific) and 1 μg used to synthesize cDNA, using the Maxima First Strand cDNA synthesis kit (Fermentas, Waltham, MA, United States). For semi-quantitative RT-PCR, *CHR8*, *UVSSA, AtCSA-1*, and *UBP12* were amplified for 30 cycles and *RDO2* for 26 cycles using the primers indicated (Supplementary Table S1) and the *Actin* loading control amplified for 22 cycles. For quantitative real time PCR, cDNA was diluted 40 fold and PCR performed using SsoFast EvaGreen Supermix (Bio-Rad, Hercules, CA, United States), a CFX Connect Real time PCR detection system (Bio-Rad), and the primers listed in Supplementary Table S1. *EF1α* (At5g60390) (Jain et al., 2006; Hossain et al., 2012) was used to normalize sample loading and three technical replicates were analyzed per sample.

### Adult Growth Analysis

The following data was collected from plants transplanted to soil: flowering time (day the first bud is detected), rosette diameter at 4 weeks, number of shoots and silique length at 6 weeks.

### UV Sensitivity Assays

Seeds were plated, stratified, and grown vertically in the conditions above for 3 days, then seedlings irradiated with 1000 J m^-2^ UV-C (corresponding to 65 s exposure to shortwave UV lamp XX-15S, UVP/LLC, Upland, CA, United States). Plates were rotated 90° and incubated in either long day or dark conditions for the indicated number of days, then scanned. Image J was used to measure root and hypocotyl length.

For adult UV assays, 21 day old plants in soil were irradiated with 500 J m^-2^ UV-C, incubated in the dark for 3 days, then returned to long day conditions. Three days later, individual leaves were scored as either undamaged (green) or damaged (yellow or brown), and % damaged leaves (number of damaged leaves/total leaves) was calculated for all plants.

### Statistical Analysis

All experiments were performed at least twice and representative experiments shown. Two-tailed student’s *t*-tests (*p* ≤ 0.05) were used to assess statistical significance.

## Results

**FIGURE 1:**
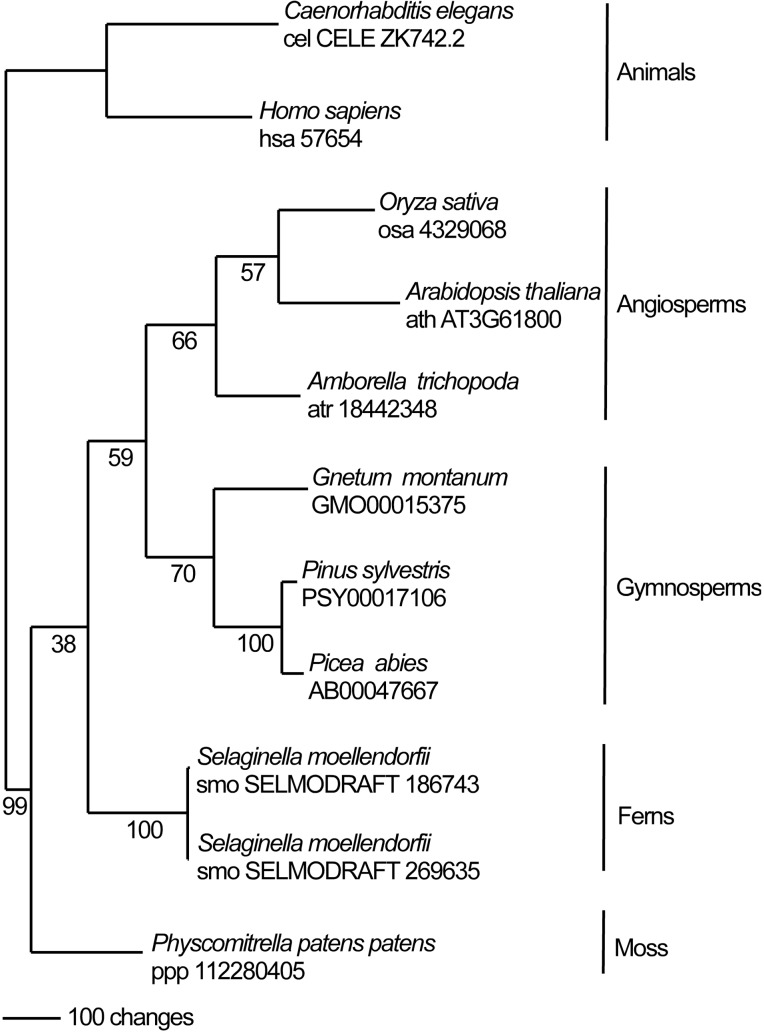
The single most parsimonious phylogenic tree (score 2561) based on analysis of amino acid sequences of UVSSA homologs. Maximum parsimony bootstrap support is indicated under each node. Gymnosperm UVSSA homologs are from the PLAZA gymnosperm site (https://bioinformatics.psb.ugent.be/plaza/versions/gymno-plaza/) (Proost et al., 2015) while all other homologs were accessed via KEGG (Kyoto Encyclopedia of Genes and Genomes) (http://www.kegg.jp/).

**FIGURE 2:**
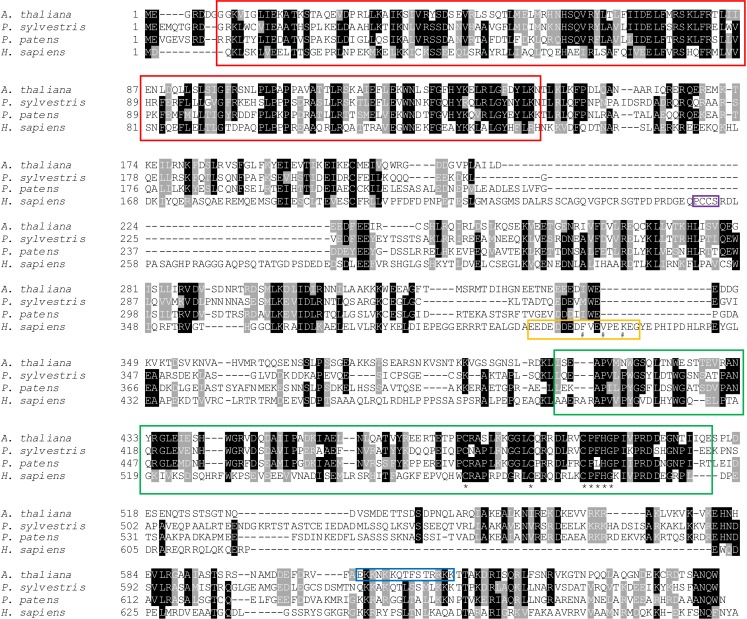
Amino acid alignment of UVSSA from representative angiosperm (*Arabidopsis thaliana*), gymnosperm (*Pinus sylvestris*), moss (*Physcomitrella patens patens*), and animal (*Homo sapiens*) species. Sequences were aligned using NCBI COBALT (Papadopoulos and Agarwala, 2007) and formatted using Boxshade. Amino acids showing identity (black) and similarity (gray) are indicated. Conserved ENTH/VHS (red) and DUF2043 (green) domains are boxed, with asterisks indicating conserved cysteines and CP(y/l)HG motif in the DUF2043 domain (Marchler-Bauer et al., 2017). The blue box indicates potential bipartite NLS in Arabidopsis UVSSA identified using Prosite (De Castro et al., 2006). Regions required for interaction of human UVSSA with USP7 (purple) and TFIIH (yellow) are shown and key residues indicated with #s (Higa et al., 2016, 2018; Okuda et al., 2017).

In this study we identify the Arabidopsis UVSSA homolog. Arabidopsis UVSSA (encoded by At3g61800) is 39% and 28% identical to rice and human UVSSA, respectively. Clear UVSSA homologs are found throughout the animal and plant kingdoms including angiosperms, gymnosperms, ferns, and moss. One million maximum parsimony phylogenetic search replicates for UVSSA homolog amino acid sequences recovered a single most parsimonious tree (score 2561) (Figure 1) that is topologically congruent with well supported hypotheses of plant evolutionary history (Morris et al., 2018). Conserved domains in UVSSA proteins include ENTH/VHS in the N terminus and DUF2043 in the C terminus (Figure 2). ENTH/VHS domains are multi-helical with an alpha-alpha 2-layered structural fold, while DUF2043 is an approximately 100 amino acid long UVSSA-specific domain, which includes three conserved cysteines and a CP(y/l)HG motif (Marchler-Bauer et al., 2017). AtUVSSA has a potential bipartite NLS in the C terminus and SUBAcon predicts nuclear localization (score 0.994) (De Castro et al., 2006; Hooper et al., 2014), consistent with a role in DNA repair.

Public gene expression data was examined for Arabidopsis *UVSSA* and the other TCR gene homologs: *AtCSA-1*, *CHR8* (*CSB* homolog), *UBP12* and *UBP13* (*USP7* homologs), and *RDO2* (*TFIIS* homolog). With respect to absolute levels of expression (Supplementary Figure S1A), *UBP12*, *UBP13*, and *RDO2* are expressed throughout the plant, consistent with the broad role of these genes in development (Grasser et al., 2009; Cui et al., 2013; Derkacheva et al., 2016), while *AtCSA-1, CHR8*, and *UVSSA* are expressed at lower levels (Schmid et al., 2005). With respect to relative levels of expression (Supplementary Figure S1B), *CHR8* and *UVSSA* are enriched in mature pollen, while *RDO2, AtCSA-1, CHR8*, and *UVSSA* are up-regulated more than two-fold in dry seed, perhaps contributing to maintenance of seed genome integrity (Waterworth et al., 2015).

Public expression data was also examined to determine the effect of potentially mutagenic stress on expression of these genes. *CHR8* was found to be upregulated by genotoxic stress induced by bleomycin and mitomycin C treatment, consistent with previous reports (Molinier et al., 2005), in both the shoot and root, but the other genes were not, while UV-B treatment did not result in major changes to the levels of any of the genes (Supplementary Figure S2; Kilian et al., 2007).

**FIGURE 3:**
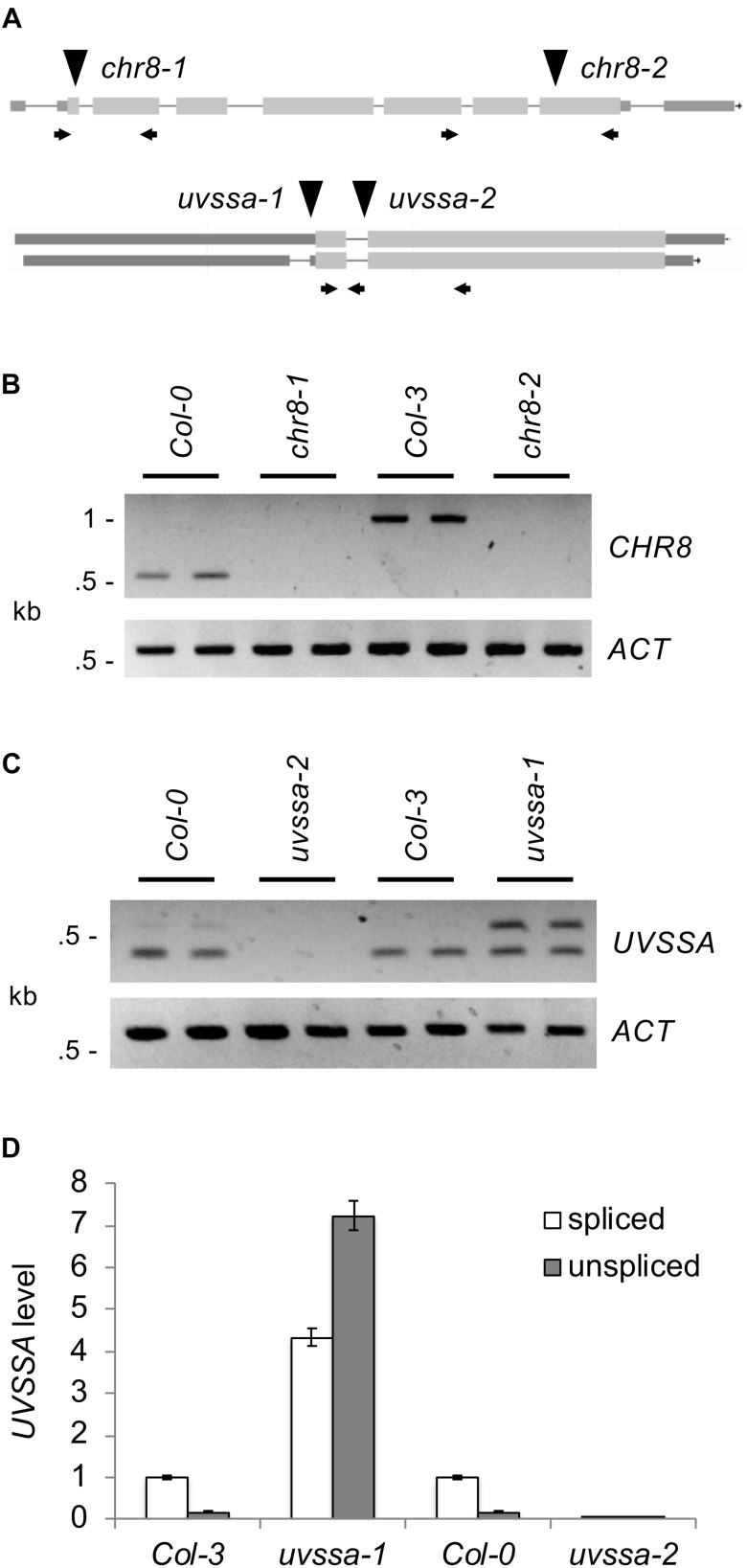
Analysis of *CHR8* and *UVSSA* alleles. **(A)** Map of *CHR8* (*At2g18760*) and *UVSSA (At3g61800)* genes. Lines indicate introns and boxes exons, with coding regions shaded light gray and untranslated regions dark gray. For *CHR8*, transcript *At2g18760.4* is shown because it is the best match to light grown seedling RNA-Seq data in JBrowse (Buels et al., 2016). For *UVSSA*, both known transcripts, *At3g61800.1* (above) and *At3g61800.2* (below), are shown. The location of T-DNA alleles *chr8-1* (SALK_000799), *chr8-2* (SAIL_273_G11), *uvssa-1* (SAIL_58_C12), and *uvssa-2* (SALK_061538) are indicated with triangles. Arrows indicate primers used in RNA analysis. **(B)** Semi-quantitative RT-PCR analysis of *CHR8* and *ACTIN* expression in *chr8-1* and *chr8-2* and their respective wild-type backgrounds, using *CHR8* primers flanking each allele. **(C)** Semi-quantitative RT-PCR analysis of *UVSSA* and *ACTIN* expression in *uvssa-1* and *uvssa-2* and their respective wild-type backgrounds, using *UVSSA* primers in the first and second coding exons. For **(B,C)**, two technical replicates per sample are shown. **(D)** qPCR analysis of spliced (primers in first coding exon and spanning exon 1–2 junction) and unspliced (primers in first coding exon and following intron) *UVSSA* level. *UVSSA* level was normalized using *EF1α* and expressed as relative to Col-3 spliced product level. Error bars indicate SE of three technical replicates.

In order to examine the role of these genes in Arabidopsis UV tolerance, T-DNA insertion mutants were obtained. Previously described alleles of *AtCSA-1* (SALK_030558) (Lee et al., 2010) and *RDO2* (SALK_027259) (Grasser et al., 2009; Liu et al., 2011) were utilized. *UBP12* allele GABI_742C10 (*ubp12-2*) has previously been shown to result in reduced levels of both *UBP12* and *UBP13*, thus acts as a weak double mutant (Cui et al., 2013). In previous studies RNAi lines of *CHR8* were shown to exhibit UV sensitivity (Shaked et al., 2006). Here we examine two T-DNA alleles of *CHR8*, *chr8-1* (SALK_000799) and *chr8-2* (SAIL_273_G11) (Figure 3A). For *UVSSA*, two T-DNA alleles were examined, *uvssa-1* (SAIL_58_C12), located 38 bp upstream of the start codon, and *uvssa-2* (SALK_061538), located in the first intron past the start codon.

We examined the effect of these alleles on gene expression using semi-quantitative RT-PCR. Primers flanking the *chr8-1* and *chr8-2* insertion sites detected no *CHR8* transcript, indicating these are null alleles (Figure 3B). Semi-quantitative RT-PCR with T-DNA insertion flanking primers also confirmed loss of transcript in the *atcsa-1, ubp12*, and *rdo2* lines (Supplementary Figure S3). For *UVSSA*, we utilized primers in the first and second coding exons, since the effect of T-DNA insertion on coding sequences was our primary concern. *uvssa-2* results in a null allele, but in *uvssa-1* both the predicted band and a larger band were detected (Figure 3C). The size of the larger band was consistent with that of the unspliced transcript, so we hypothesized that *uvssa-1* insertion affected intron splicing [note the *uvssa-1* samples did not result in larger gDNA-size bands of *CHR8*, thus were not gDNA contaminated (data not shown)]. Real-time qPCR with an intron-specific primer was used to quantify the effect of the *uvssa-1* allele on splicing, and large amounts of the unspliced product were detected (Figure 3D). Due to the presence of an in frame stop codon in the intron, this transcript results in a truncated 77 amino acid product. *uvssa-1* also resulted in increased levels of correctly spliced *UVSSA*. Thus *uvssa-1* would be predicted to result in increased levels of both full length and truncated UVSSA.

Mutant alleles of the TCR genes were grown in long day conditions with their respective controls and their developmental phenotypes examined. *ubp12-2* mutants exhibited decreased rosette size, early flowering (days), and decreased apical dominance (increased number of shoots) (Supplementary Figure S4), consistent with previously described phenotypes (Cui et al., 2013; Derkacheva et al., 2016). The other mutant alleles did not exhibit any developmental phenotypes with the exception of a slight increase in apical dominance in *chr8-2*. *RDO2* mutants have been described as early flowering (Grasser et al., 2009), however, additional analysis indicates this phenotype is observed with respect to number of leaves, rather than number of days, at flowering (Mortensen and Grasser, 2014), consistent with our results.

**FIGURE 4:**
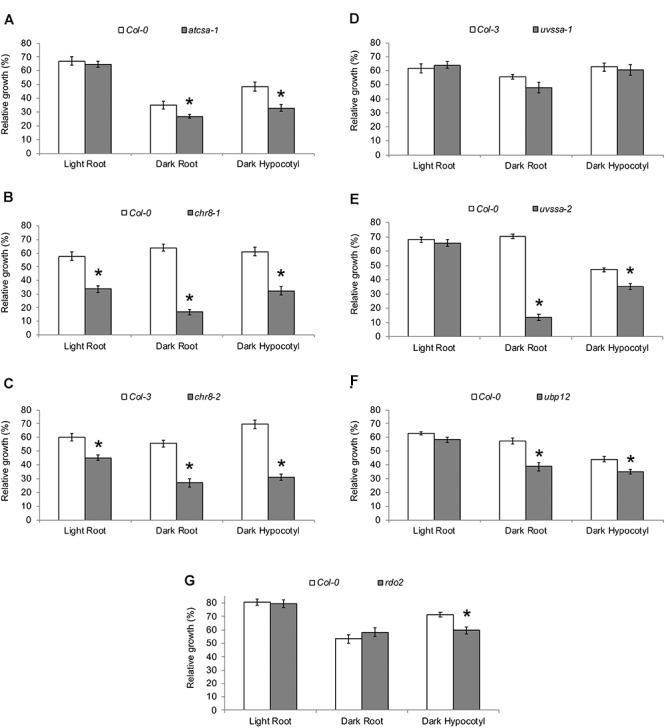
UV tolerance of mutants in TCR genes. Relative growth of roots and hypocotyls of **(A)** *atcsa-1*, **(B)** *chr8-1*, **(C)** *chr8-2*, **(D)** *uvssa-1*, **(E)** *uvssa-2*, **(F)** *ubp12*, and **(G)** *rdo2* after 1000 J m^-2^ UV treatment, followed by 2 days of long-day (light) or dark incubation. Data are expressed as length relative to unirradiated control of the same genotype. Values are means ± SE (*n* = 20), ^∗^*p* ≤ 0.05 of mutants vs wild type.

**FIGURE 5:**
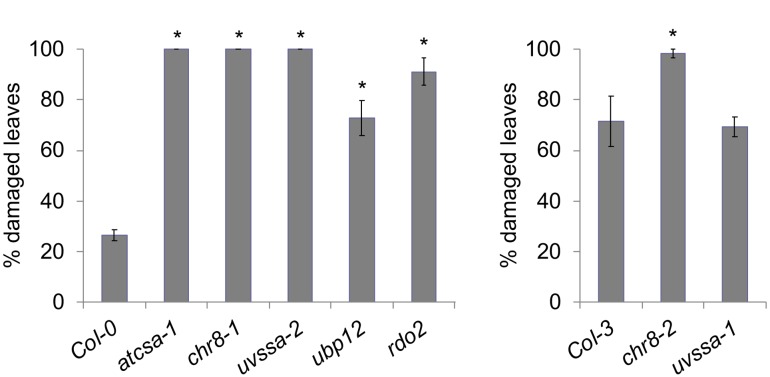
UV tolerance in adult plants. Percentage damaged leaves after 500 J m^-2^ UV treatment, followed by 3 days of dark incubation. Values are means ± SE (*n* = 6), ^∗^*p* ≤ 0.05 of mutants vs respective wild type.

The UV tolerance of the mutant alleles of the TCR genes was then assessed. Since TCR is a sub-pathway of NER, or dark repair, we assessed UV tolerance in seedlings following dark incubation after UV treatment. As previously described (Shaked et al., 2006; Biedermann and Hellmann, 2010; Zhang et al., 2010), *AtCSA-1* and *CHR8 (CSB)* loss of function resulted in increased UV sensitivity in the dark (Figure 4A–C and Supplementary Figures S5A,B). The *UVSSA* loss of function allele, *uvssa-2*, also resulted in increased UV sensitivity in the dark (Figure 4E). The *uvssa-1* allele, which results in increased levels of both truncated and full length UVSSA, did not exhibit either increased or decreased UV tolerance following 2 or 3 days of dark incubation (Figure 4D and Supplementary Figure S5C). *ubp12-2* also exhibited increased UV sensitivity in the dark (Figure 4F and Supplementary Figure S5D). *rdo2* exhibited increased dark UV sensitivity in hypocotyls (but not roots) after 2 days of incubation, but not after 3 days (Figure 4G and Supplementary Figure S5E). We also examined UV sensitivity in adult plants following dark incubation and found that, as in seedlings, *atcsa-1, chr8, uvssa-2, ubp12*, and *rdo2* mutants exhibit UV sensitivity, while *uvssa-1* does not (Figure 5).

To examine the specificity of the UV sensitivity of these alleles, they were also incubated in light (long day) following UV treatment. *atcsa-1, uvssa-2*, and *ubp12* were not UV sensitive in the light (Figure 4A,E,F), consistent with the dark specific role of NER. Surprisingly, both *chr8* alleles displayed UV sensitivity following light incubation (Figure 4B,C), exhibiting the expected dose dependence, with the more severely truncated *chr8-1* allele demonstrating a stronger root phenotype in both light and dark. This result suggests that CHR8 plays a role in light repair, distinct from the other components of the TCR pathway.

## Discussion

In this study, we examined the UV sensitivity of mutant alleles of Arabidopsis homologs of genes implicated in mammalian TCR. As previously reported, we find *atcsa-1* mutants exhibit increased dark specific UV sensitivity (Biedermann and Hellmann, 2010). Our *atcsa-1* dark root phenotype is not as strong as that of mutants in other TCR components such as CSB/CHR8 and UVSSA, this may be due to redundancy with AtCSA-2/CSAat1B.

The Arabidopsis homolog of mammalian CSB [also known as Excision Repair Cross-Complementing 6 (ERCC6)] and yeast Rad26 is CHR8 (Kunz et al., 2005; Singh et al., 2010). In this study, we utilized *CHR8* T-DNA lines and observed increased UV sensitivity following dark incubation, consistent with previous studies using *CHR8* RNAi lines (Shaked et al., 2006). Also, unique among the TCR mutants we examined, *chr8* alleles exhibited increased UV sensitivity following light incubation. Mammalian CSB has been implicated in regulation of transcription and base excision repair in addition to TCR (Stevnsner et al., 2008; Boetefuer et al., 2018), so one of these roles may contribute to the *chr8* light UV sensitivity phenotype.

In humans, mutation of *UVSSA* results in defective TCR and UV sensitive syndrome (Cleaver, 2012). Loss of the *C. elegans* UVSSA homolog also results in increased UV sensitivity (Babu and Schumacher, 2016). While UVSSA is conserved throughout the animal kingdom (Nakazawa et al., 2012), it is absent from Drosophila. However, Drosophila also lack CSA and CSB homologs, and do not appear to perform TCR (Sekelsky, 2017). Yeast also lack UVSSA, although both *S. cerevisiae* and *S. pombe* have CSB homologs and perform TCR (Li and Li, 2017; Xu et al., 2017). Here we show that UVSSA is found throughout the plant kingdom, with conserved ENTH/VHS and DUF2043 domains. Recently, the region corresponding to amino acid 400–415 of human UVSSA was found to be well conserved in animals and required for TFIIH interaction (Okuda et al., 2017). Although this region is still acidic in plants, it not well conserved with human UVSSA and plants lack F408 and V411, which are required for TFIIH interaction and TCR in humans (Okuda et al., 2017), as well as K414, which is mono-ubiquitinated (Higa et al., 2018). In addition, residues 251–254 of human UVSSA have been shown to be required for USP7 interaction, CSB stability, and TCR (Higa et al., 2016), yet this sequence is also not conserved in plants. Nonetheless our data show that lack of UVSSA results in dark specific UV sensitivity in Arabidopsis, consistent with a role in NER.

Arabidopsis USP7 homologs UBP12 and UBP13, like other ubiquitin specific proteases, play important roles in plant development and environmental response (Zhou et al., 2017). UBP12/13 interact with LHP1 and deubiquitinate RGFR1 and MYC2 (Derkacheva et al., 2016; Jeong et al., 2017; An et al., 2018). Our results here indicate that UBP12 (and UBP13) are involved in UV tolerance, suggesting they may also deubiquitinate UVSSA and CSB, as has been proposed for mammalian USP7 (Geijer and Marteijn, 2018). UBP12 and UBP13 act redundantly, and double null alleles are inviable due to pollen defects (Ewan et al., 2011; Derkacheva et al., 2016). Here we use an allele of *UBP12, ubp12-2*, which also results in a partial decrease in *UBP13* level, and resulting in a weak double mutant (Cui et al., 2013). However, because this is a weak (non-null) double mutant, we may be underestimating the role of UBP12/13 in UV tolerance.

In mammals, in addition to acting during transcript elongation, TFIIS has been shown to facilitate transcription re-initiation following RNAP arrest, and is recruited to the stalled polymerase in a CSB and CSA dependent manner (Donahue et al., 1994; Kalogeraki et al., 2005; Fousteri et al., 2006; Dutta et al., 2015). In yeast, loss of TFIIS only results in increased UV sensitivity in a GGR-deficient background, however, the same is true of CSB homolog Rad26 (Wong and Ingles, 2001). In mammals, reduction of TFIIS resulted in reduced RNA synthesis recovery, but had no effect on UV sensitivity (Jensen and Mullenders, 2010). In this study we detected a UV sensitive phenotype in TFIIS deficient Arabidopsis (*rdo2*), however, it was milder than observed for the other TCR mutants and not detectable 3 days after seedling UV treatment. Interestingly, the UV sensitive phenotype of both *rdo2* and *atcsa-1* was stronger in hypocotyls than in roots, at 2 days than at 3 days, and in adults than in seedlings, suggesting the role of these genes in UV tolerance may vary with tissue, time, and phenotype assessed (growth versus tissue death).

## Conclusion

In this study, we have identified the Arabidopsis UVSSA homolog and shown that Arabidopsis UVSSA, USP7 (UBP12/13), and TFIIS (RDO2) homologs contribute to UV tolerance, along with CSA and CSB (CHR8) homologs, suggesting conservation in the mechanisms of TCR.

## Data Availability

All datasets for this study are included in the manuscript and the Supplementary Files.

## Author Contributions

WAK, AS, and DS performed the experiments. JM conducted the phylogenetic analysis. DS wrote the first draft of the manuscript. All authors contributed to revised manuscript and approved the final version, designed the experiments and analyzed the data.

## Conflict of Interest Statement

The authors declare that the research was conducted in the absence of any commercial or financial relationships that could be construed as a potential conflict of interest.

## Footnotes

1 https://bioinformatics.psb.ugent.be/plaza/versions/gymno-plaza/

2 http://www.kegg.jp/

## Abbreviations

CHR8: chromatin remodeling 8
CSA/B: Cockayne syndrome A/B
GGR: global genomic repair
NER: nucleotide excision repair
NER: nucleotide excision repair
RDO2: reduced dormancy 2
RNAP: RNA polymerase II
TCR: transcription coupled repair
TFIIS: transcription elongation factor IIS
UBP and USP: ubiquitin specific protease
UV: ultraviolet irradiation
UVSSA: UV stimulated scaffold protein A
