# Supplementary material for: UVSSA, UBP12, and RDO2/TFIIS Contribute to Arabidopsis UV Tolerance
Source: Front Plant Sci. 2019 Apr 24;10:516. doi: 10.3389/fpls.2019.00516 (PMC6492544; doi:10.3389/fpls.2019.00516)
Supplement: Supplementary file 1 [file Data_Sheet_1.pdf]

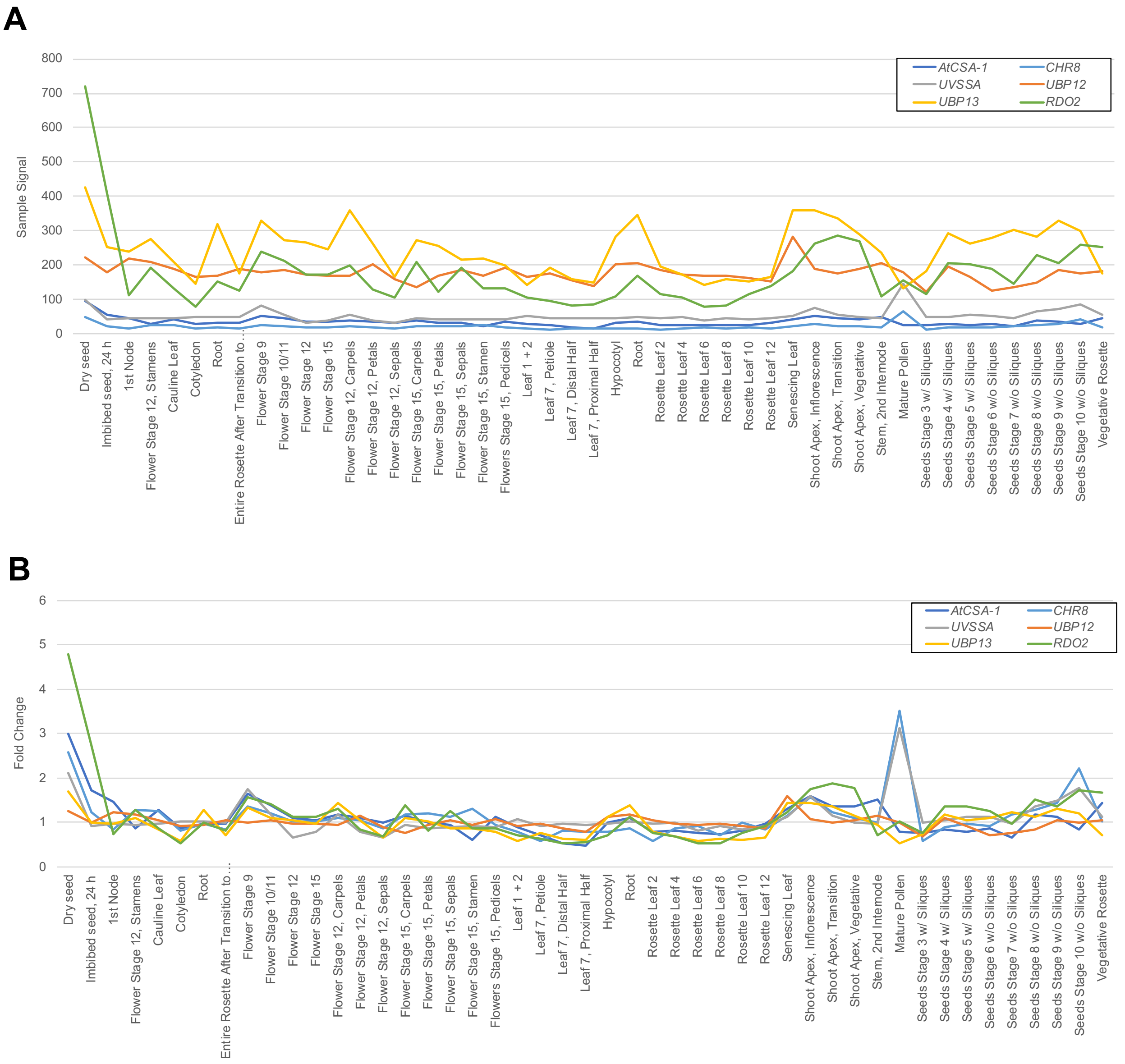

Supplementary Figure S1. Expression of Arabidopsis transcription coupled repair genes throughout development. Data from Schmid et al. (2005) accessed via the Arabidopsis eFP browser (Winter et al., 2007). Absolute **(A)** and relative **(B)** expression are shown.

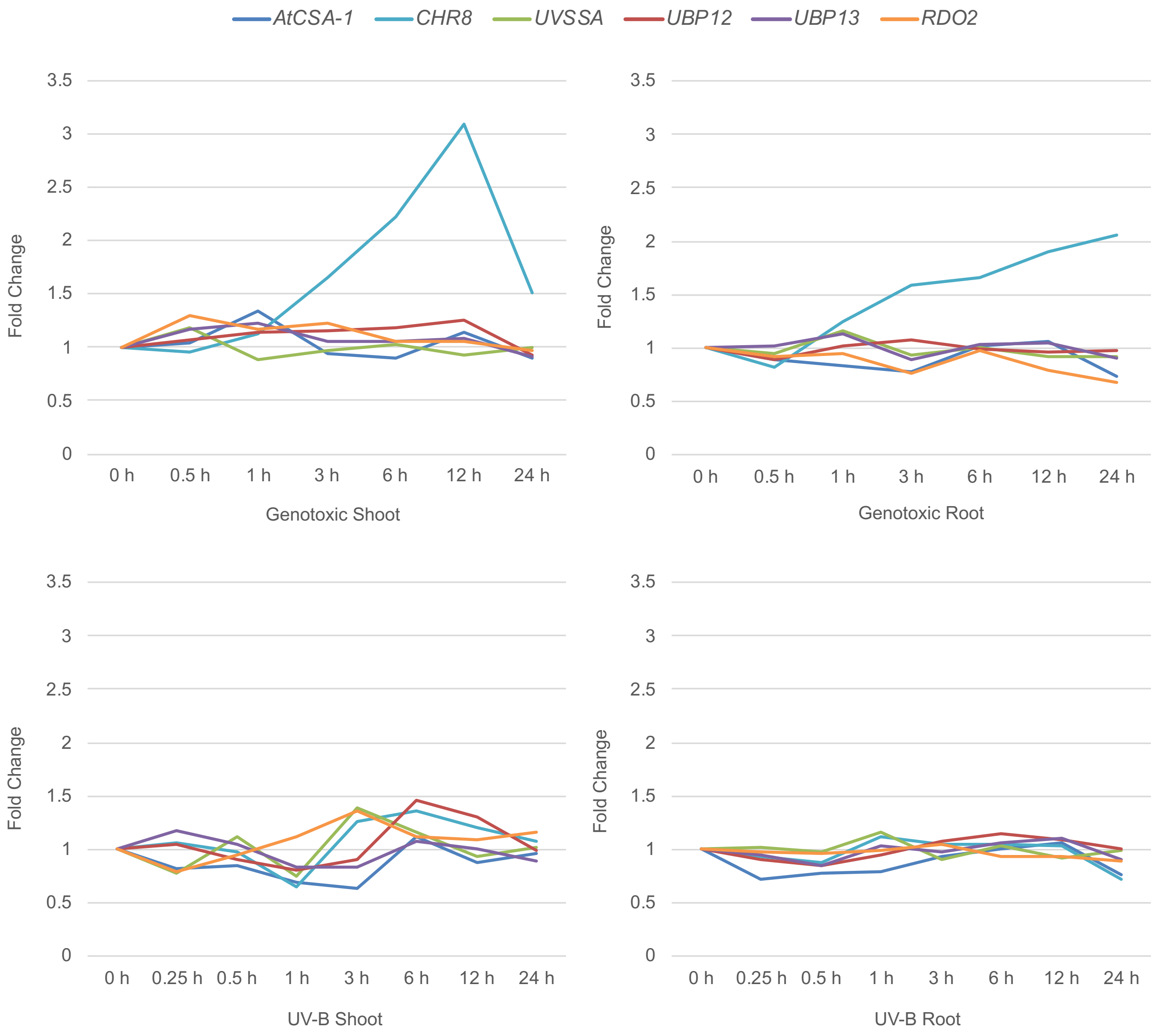

Supplementary Figure S2. Expression of Arabidopsis TCR genes following potentially mutagenic stress. Relative level of TCR genes in response to genotoxic (bleomycin 1.5 g/ml plus mitomycin C 22 g/ml) (upper panels) and UV-B (15 min UV-B derived from six 40W/12 UV fluorescent tubes filtered through transmission cutoff filters) (lower panels) stress. Expression in aerial (shoot) (left panels) and root (right panels) tissues is shown. Data from Kilian et al. (2007) accessed via AtGenExpress.

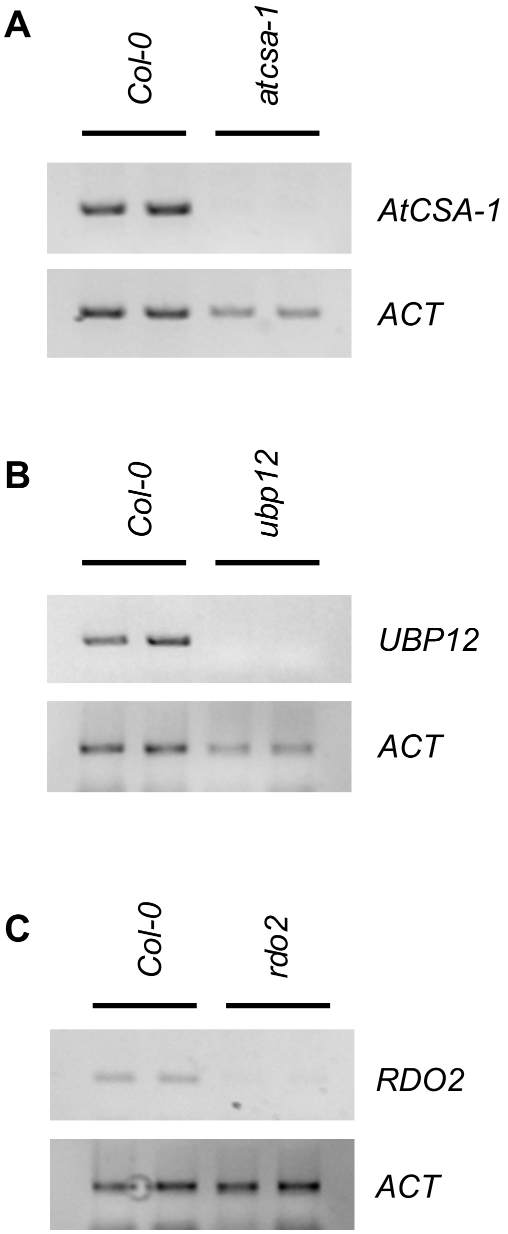

Supplementary Figure S3. Semi-quantitative RT-PCR analysis of *atcsa-1* **(A),** *ubp12* **(B)**, and *rdo2* **(C)** alleles with primers flanking respective T-DNA insertions, versus *ACTIN* loading control. Two technical replicates are shown.

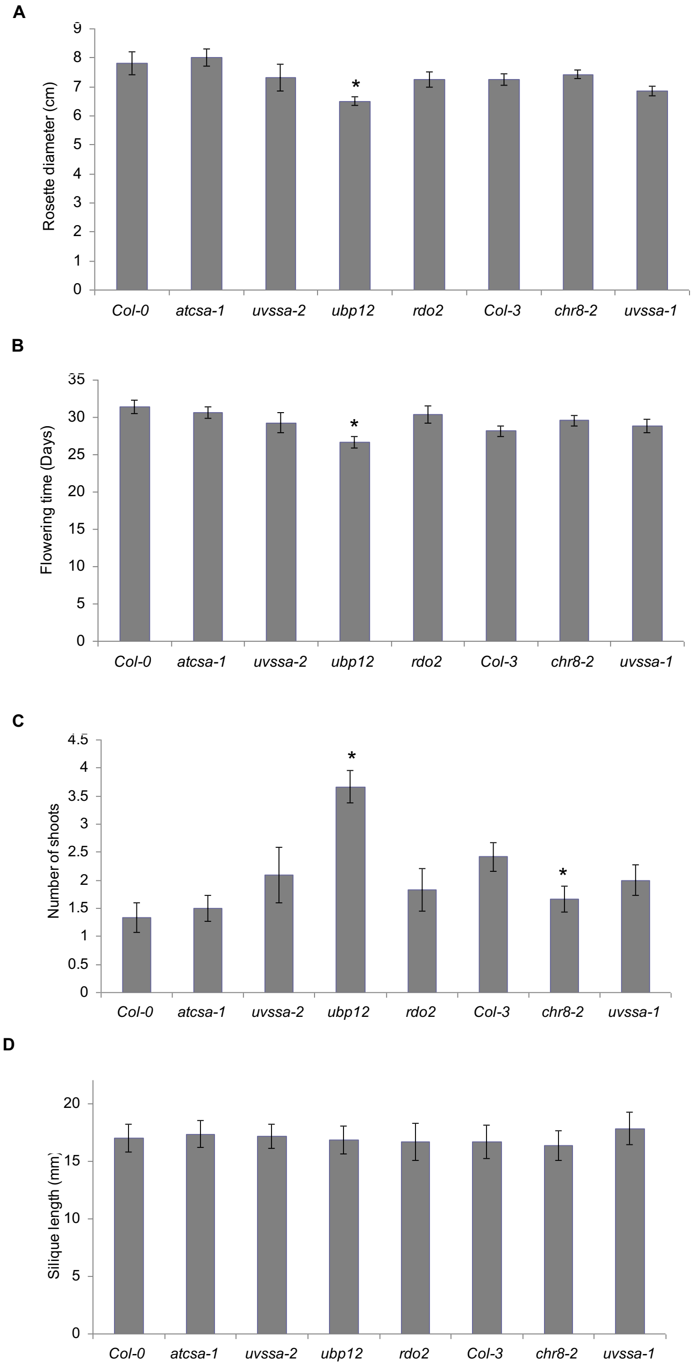

Supplementary Figure S4. Developmental phenotypes of mutants in TCR genes. *atcsa-1, uvssa-2, ubp12,* and *rdo2* are in the Col-0 background while *chr8-2* and *uvssa-1* are in the Col-3 background. **(A)** Rosette diameter at 4 weeks. **(B)** Flowering time (days). **(C)** Apical dominance (number of shoots) at 6 weeks. **(D)** Silique length at 6 weeks. Values are means ± SE (n= 6). * = $p \leq 0.05$ of mutant vs respective wild type.

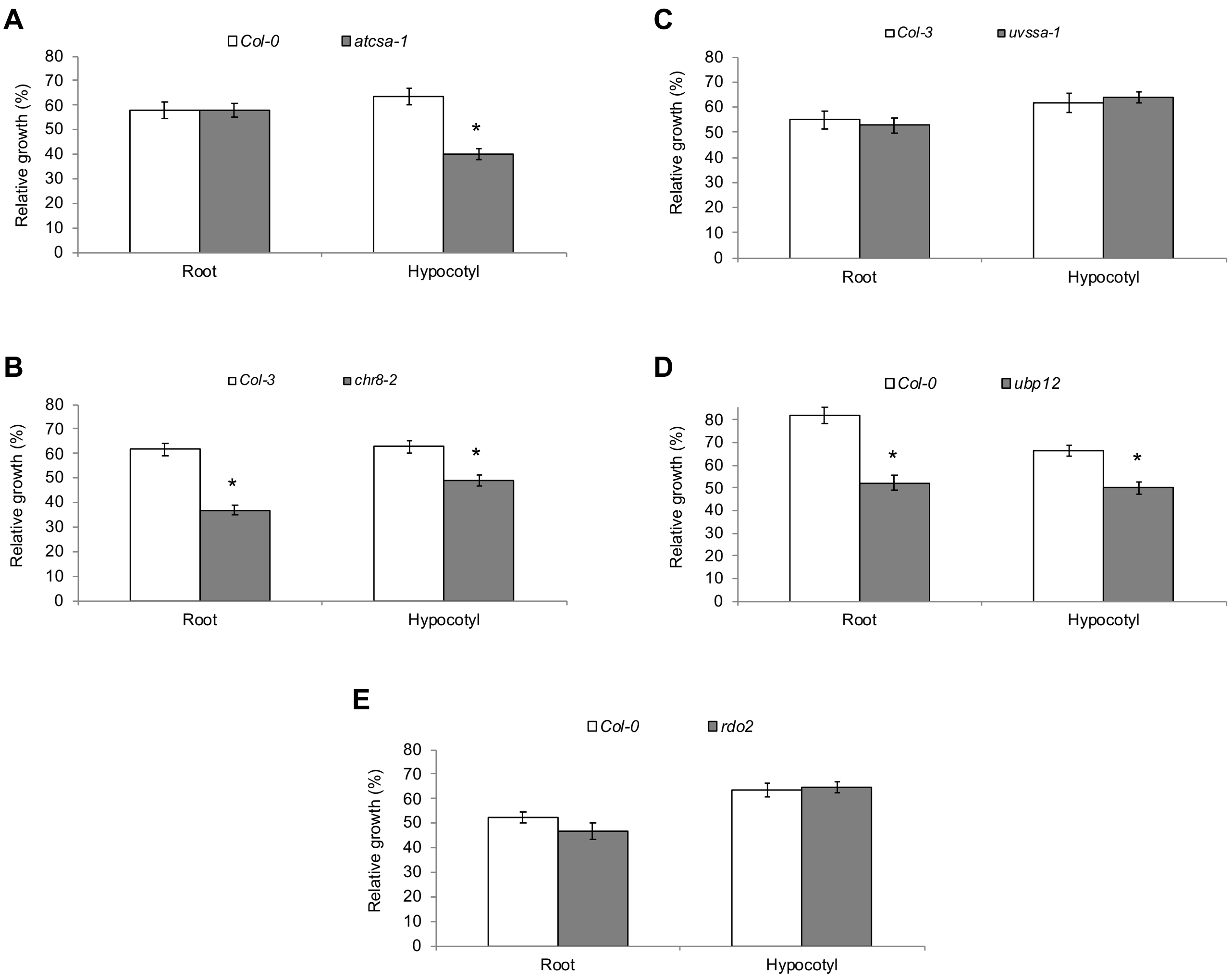

Supplementary Figure S5. UV tolerance of mutants in TCR genes. Relative growth of roots and hypocotyls after 1000 J m^{-2} UV treatment, followed by 3 days of dark incubation. Data are expressed as length relative to unirradiated control of the same genotype. Values are means ± SE (n = 20), * = $p \leq 0.05$ of mutants vs wild type.

Supplementary Table S1. Primers used in this study.

Genotyping				
CSA	CSA-1L	TGTGGATTTTAAAATGCCTGG	CSA-1R	CCAGCAGATGCTGCCTATAAC
CHR8	CSB-1L	TACCGTTTCAACAAAACCAGC	CSB-1R	TCTTTGACGAAACCAGTTTCG
	CSB-2L	ATTATCTTTGACCCGGATTGG	CSB-2R	CGTGGCAATCTCTTTCAAGAG
UVSSA	UVSSA-1L	TCTGGTGGTGGTAGGTTTGAG	UVSSA-1R	TCAATAGCCTTTGAACGCAAC
	UVSSA-2L	GAGCAAGAAGCCATTGAGATG	UVSSA-2R	CTGTCTCTCTCGTTGAATCCG
UBP12	UBP12-2L	TTCATGTTTTGGGGCTAATTG	UBP12-2R	TGGTATGCCTTGCAGATTTTC
RDO2	RDO2-2L	CACATACGATACCCTCGTTGC	RDO2-2R	GGTAGATGCTTTTGCAGCAAG
Semi-quantitative RT-PCR				
CSA	CSA-1L	TGTGGATTTTAAAATGCCTGG	CSA_c1083R	ACATCCCATCGTTAGTTGCC
CHR8	CSB_c45F	GACATCGGCCAATCCTGAAG	CSB-1L	TACCGTTTCAACAAAACCAGC
	CSB-2L	ATTATCTTTGACCCGGATTGG	CSB-2R	CGTGGCAATCTCTTTCAAGAG
UVSSA	UVSSA_c76F	GTTGACCCGAGGCTTCTCAA	UVSSA-2R	CTGTCTCTCTCGTTGAATCCG
UBP12	UBP12_c3108F	GCATCCAGATGCAGAACTGA	UBP12-2L	TTCATGTTTTGGGGCTAATTG
RDO2	RDO2_c637F	TCAGAGGGGTGATTCGGCTA	RDO2-2R	GGTAGATGCTTTTGCAGCAAG
ACTIN	ACT F	CTGGAACAAGACTTCTGGGC	ACT R	GGTGATGAAGCACAATCCAAG
qPCR				
UVSSA	UVSSA_c76F	GTTGACCCGAGGCTTCTCAA	UVSSA_c198R	CAGATATCGAACCTGAGAGTGGT
	UVSSA_c129F	GGAGGTCCGACTTAGTTCGC	UVSSA_In298R	CACCGCGTTATTTGACCAACA
EF1α	EEF F	CTGGAGGTTTTGAGGCTGGTAT	EEF R	CCAAGGGTGAAAGCAAGAAGA

Supplementary References

Kilian, J., Whitehead, D., Horak, J., Wanke, D., Weinl, S., Batistic, O., et al. (2007) The AtGenExpress global stress expression data set: protocols, evaluation and model data analysis of UV-B light, drought and cold stress responses. *Plant J.* 50, 347-363.

Schmid, M., Davison, T. S., Henz, S. R., Pape, U. J., Demar, M., Vingron, M., et al. (2005) A gene expression map of *Arabidopsis thaliana* development. *Nat. Genet.* 37, 501-506.

Winter, D., Vinegar, B., Nahal, H., Ammar, R., Wilson, G. V., and Provart, N. J. (2007) An "Electronic Fluorescent Pictograph" browser for exploring and analyzing large-scale biological data sets. *PLoS One* 2, e718.
